# Magnetron Sputtering of Polymeric Targets: From Thin Films to Heterogeneous Metal/Plasma Polymer Nanoparticles

**DOI:** 10.3390/ma12152366

**Published:** 2019-07-25

**Authors:** Ondřej Kylián, Artem Shelemin, Pavel Solař, Pavel Pleskunov, Daniil Nikitin, Anna Kuzminova, Radka Štefaníková, Peter Kúš, Miroslav Cieslar, Jan Hanuš, Andrei Choukourov, Hynek Biederman

**Affiliations:** 1 Department of Macromolecular Physics, Faculty of Mathematics and Physics, Charles University, V Holešovičkách 2, 180 00 Prague 8, Czech Republic; 2 Department of Surface and Plasma Science, Faculty of Mathematics and Physics, Charles University, V Holešovičkách 2, 180 00 Prague 8, Czech Republic; 3 Department of Physics of Materials, Faculty of Mathematics and Physics, Charles University, Ke Karlovu 5, 121 16 Prague 2, Czech Republic

**Keywords:** magnetron sputtering, nanoparticles, gas aggregation sources

## Abstract

Magnetron sputtering is a well-known technique that is commonly used for the deposition of thin compact films. However, as was shown in the 1990s, when sputtering is performed at pressures high enough to trigger volume nucleation/condensation of the supersaturated vapor generated by the magnetron, various kinds of nanoparticles may also be produced. This finding gave rise to the rapid development of magnetron-based gas aggregation sources. Such systems were successfully used for the production of single material nanoparticles from metals, metal oxides, and plasma polymers. In addition, the growing interest in multi-component heterogeneous nanoparticles has led to the design of novel systems for the gas-phase synthesis of such nanomaterials, including metal/plasma polymer nanoparticles. In this featured article, we briefly summarized the principles of the basis of gas-phase nanoparticles production and highlighted recent progress made in the field of the fabrication of multi-component nanoparticles. We then introduced a gas aggregation source of plasma polymer nanoparticles that utilized radio frequency magnetron sputtering of a polymeric target with an emphasis on the key features of this kind of source. Finally, we presented and discussed three strategies suitable for the generation of metal/plasma polymer multi-core@shell or core-satellite nanoparticles: the use of composite targets, a multi-magnetron approach, and in-flight coating of plasma polymer nanoparticles by metal.

## 1. Introduction

Sputtering is a deposition process based on the ejection of atoms, molecules, or molecular fragments from a target that is bombarded by energetic particles (mostly ions) and subsequent condensation of emitted particles on adjacent surfaces. Since its discovery in the mid-1800s (for the history of sputtering, please refer to J.E. Green’s excellent recent review [1]), sputtering has become one of the most widely used techniques for film deposition, with thickness reaching from several nm to several μm. Despite the long history of sputter deposition, the introduction of external magnetic fields was a crucial moment that led to a massive spread of sputtering technology. Specifically, a configured magnetic field constrains the plasma to a close proximity to the cathode and enormously increases the deposition rate. Among the different configurations that are altogether termed as “magnetrons”, systems with a planar configuration appear to be the most important [2]. Since their introduction, such planar magnetrons have become irreplaceable tools for the deposition of various conductive, mostly metallic, thin films. 

In addition, the demand for the production of coatings with enhanced functional properties and deposition of non-metallic/non-conductive thin films triggered the development of novel concepts of magnetron sputtering. These include high-power pulsed magnetron sputtering [3,4,5,6,7], dual-magnetron sputtering [8], and radio frequency (RF) magnetron sputtering of non-conductive targets [9]. Concerning the latter, RF magnetron sputtering was found to be suitable not only for the production of inorganic materials (e.g., glasses, metal-oxides, and nitrides) but also for the deposition of polymer-like coatings [10,11,12], i.e., the so-called plasma polymers [13,14,15,16]. As opposed to conventional polymers, such materials are characterized by considerably higher levels of cross-linking and branching, as well as by an absence of regularly repeating monomer units. Despite their random and inherently complex structure, plasma polymers appear to be a highly valuable class of materials for different applications, including dielectric separation layers, permeation barriers or gas separation membranes, laser facilities, adhesion-promoting coating, and films that enable the fine tuning of wettability and the bio-adhesive/bio-repellent behavior of surfaces [17,18,19,20,21,22,23,24,25,26,27,28,29,30,31,32,33,34,35,36,37,38]. The great advantage of RF magnetron sputtering over commonly used plasma-enhanced chemical vapor deposition is the complete lack of gaseous or liquid precursors, which makes RF sputtering a “green” technology. Although much attention has been devoted to the fabrication and characterization of fluorocarbon plasma polymers [10,11,12,39,40,41,42,43,44,45,46,47], other polymers were also studied, including polyarylates [48], polyimides [45,49,50,51,52], polyethylene [50,53,54], polyetherimide [55], polypropylene [56,57], and Nylon [58].

Regardless of the sputtered material, magnetron-based deposition was primarily applied for the production of thin compact films for a long time. The situation changed in the 1990s, when Haberland and his co-workers introduced the first magnetron-based Gas Aggregation cluster Source (GAS) [59,60], which opened a completely new and highly attractive application field for magnetron sputtering technology. In this type of source, magnetron sputtering serves as a supply of supersaturated vapors that spontaneously nucleate and form clusters or nanoparticles (NPs) in the volume of the aggregation chamber at appropriate conditions (higher pressure). NPs are subsequently transferred by a carrier gas (typically argon) through a small aperture from the aggregation chamber of the GAS to the main deposition chamber, where they are collected on substrates. This deposition strategy offers several key benefits as compared to other methods used for the production of NPs—high purity, possibility to tailor kinetic energy and size distribution of produced NPs, directionality of the deposition process, which is suitable for the production of patterned surfaces, as well as the possibility to deposit NPs on the substrates of virtually any material that is compatible with high vacuum conditions. Furthermore, gas aggregation cluster sources can be easily combined with other vacuum-based deposition techniques and, hence, nanocomposite coatings with different architectures can be fabricated. For instance, our group recently reported on the fabrication of Ag/a-C:H and Cu/a-C:H nanocomposites with metallic NPs randomly distributed in the a-C:H matrix [61,62], metal/plasma polymer sandwich structures [63], gradient coatings [64,65], and multi-layered metal/plasma polymer nanocomposites [66,67,68]. 

In analogy to “conventional” magnetron sputtering, magnetron-based GAS systems were initially employed generally for the production of various metallic (e.g., Ag [69,70,71], Cu [72,73], Al [74], Ti [75,76,77], Co [78,79], Pt [80,81], Nb [82], Pd [83], W [84], Ni [85], Ru [86]) and metal-oxide NPs [87,88]. However, our group recently showed that GAS systems may also be easily adapted for the production of plasma polymer NPs if the polymeric target is sputtered in the RF mode [89,90,91,92,93]. 

Lately, the interest in the production and utilization of heterogeneous multi-component NPs led to the development of three principal approaches:

(1) Use of bi-metallic composite targets. In this case, the targets composed of two metals were sputtered, which gave rise, depending on the operational conditions, to the formation of heterogeneous NPs with different structures (core@shell, onion-like structure, dumbbell-like structure) [94,95,96].
(2) Multi-magnetron approach. Up to three individual planar magnetrons were placed into a single aggregation chamber. Depending on the mutual position of the magnetrons, the applied magnetron currents and the sputtered materials, alloy, core-satellite, Janus-like, core@shell or core@shell@shell NPs were produced [97,98,99,100,101].
(3) In-flight coating/modification of NPs. In this case, NPs produced by GAS were modified/coated in-flight in an auxiliary chamber located in between the GAS and the substrate. This method was reported to be effective for oxidation of the surface layer of metallic NPs [102], production of core@shell NPs [103,104,105,106], and NPs decorated by other materials (so-called strawberry-like or core-satellite structures) [107].

The above-mentioned strategies were successfully tested by different research groups. However, the majority of the research published to date focused on the production of inorganic multi-component NPs. The aim of this featured article is to demonstrate that all three strategies are also applicable for the production of metal/plasma polymer NPs. To meet this general aim, all the examples involved sputtered Nylon (C:H:N:O) particles only. Nylon was selected on the basis of previous studies of sputter deposition of C:H:N:O plasma polymer thin films, which revealed that such materials are suitable for various bio-medical applications as they can enhance the adhesion and attachment of biomolecules or cells [108,109], and can be utilized for the design of systems for controlled drug-delivery [110]. The article is organized as follows: Section 2 briefly presents the deposition setups; Section 3.1 presents the main features for the production of C:H:N:O NPs by the GAS source with the magnetron equipped with the Nylon 6,6 target; Section 3.2, Section 3.3, and Section 3.4 present the first results that were obtained by the use of the composite target, the dual-magnetron system, and the deposition setup for the in-flight deposition of silver onto C:H:N:O NPs; and finally, the results are briefly summarized in Section 4.

## 2. Materials and Methods 

**Figure 1:**
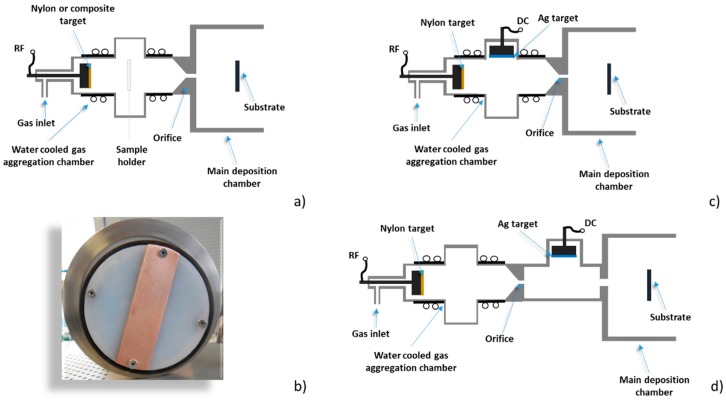
(**a**) Basic system for the deposition of plasma polymer nanoparticles (NPs). (**b**) Image of Nylon 6,6 with Cu strip. (**c**) Dual-magnetron system. (**d**) Setup for in-flight modification of C:H:N:O NPs by silver.

The system schematically depicted in Figure 1a was used for the deposition of plasma polymer C:H:N:O nanoparticles and heterogeneous metal/plasma polymer particles. It was based on a planar water-cooled RF magnetron that was inserted into a water-cooled gas aggregation chamber (102 mm inner diameter). The magnetron was equipped either by the Nylon 6,6 target (Goodfellow, 81 mm in dimeter, 3 mm thick) or by the same target with a strip of a Cu plate (Figure 1b). The magnetron was powered by an RF power supply (Dressler, Cesar 133) through an automatic matching network (ADTEC, AMV-1000-EN). If not specified elsewhere in the text, Ar was used as the working gas. The aggregation chamber was terminated by a conical lid with a circular orifice 2.5 mm in diameter. The orifice separated the aggregation chamber from the rest of the deposition system. The deposition rate was measured by a quartz crystal microbalance located in the main deposition chamber. 

In addition to the basic configuration, two modifications were tested as well. In the first case, a second magnetron equipped with an Ag target (Safina, 3 inch in diameter, with a thickness of 3 mm) was installed into the aggregation chamber perpendicularly to the one equipped with the Nylon 6,6 target (Figure 1c). This additional magnetron was operated in a direct current (DC) mode (Advanced energy, MDX 500). The second configuration was used for the in-flight coating of C:H:N:O nanoparticles by silver. In this case, an additional chamber was introduced between the GAS and the main deposition chamber (Figure 1d). This part of the deposition system consisted of 3 inch planar magnetron installed perpendicularly to the direction of the beam of the C:H:N:O nanoparticles and was ended by an orifice (3 mm in diameter). 

The morphology of the produced particles was evaluated by means of a scanning electron microscopy (SEM, MIRA 3 Tescan, Brno, Czech Republic) or a transmission electron microscopy (TEM, JEOL2200FS, Akishima, Japan). The optical properties of the fabricated nanoparticles were determined by UV-Vis spectrophotometry (Hitachi U-2910, Tokyo, Japan) in the spectral range 325 nm–800 nm.

## 3. Results

### 3.1. Gas-Phase Fabrication of C:H:N:O Nanoparticles

**Figure 2:**
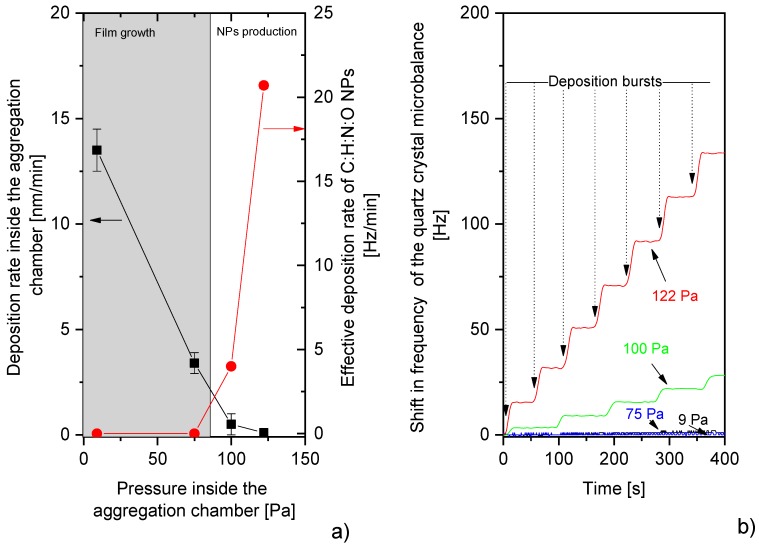
(**a**) Pressure dependences of the deposition rate of C:H:N:O film inside the aggregation chamber and effective deposition rate of C:H:N:O NPs in the main deposition chamber. (**b**) Pressure dependences of frequency shift on quartz crystal microbalance (QCM) installed into the main deposition chamber. The change in the frequency of quartz crystal is directly proportional to the deposited mass. RF power 40 W.

**Figure 3:**
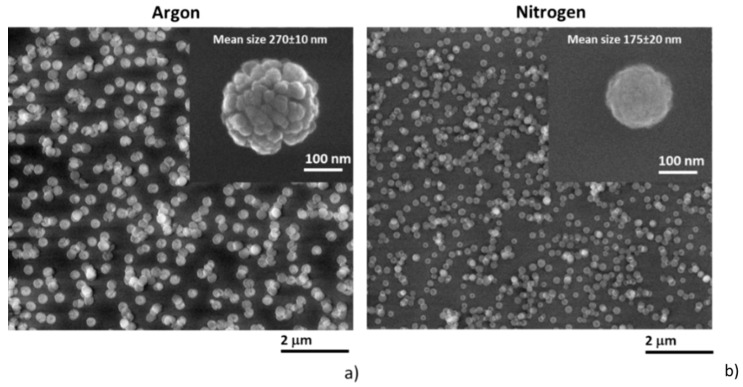
Scanning electron microscopy (SEM) images of C:H:N:O NPs deposited in (**a**) pure Ar or (**b**) N_2_. The presented mean sizes of produced NPs were evaluated from the diameters of 300 individual NPs.

The first step in this study was to investigate the properties of C:H:N:O NPs produced by the GAS system equipped with the Nylon 6,6 target. It was found that the key parameter for the production of C:H:N:O NPs was the pressure in the aggregation chamber. For low pressures, the molecular fragments emitted from the Nylon target predominantly condensed on the walls of the aggregation chamber, where they formed a thin compact film, and no NPs were detected in the main deposition chamber. The formation of thin film in the aggregation chamber was confirmed by ellipsometric measurements of the coatings deposited on Si wafers that were introduced into the aggregation chamber of the GAS at a distance of approximately 50 mm from the magnetron target. As the pressure in the aggregation chamber increased, the deposition rate of the C:H:N:O film gradually decreased and at about 100 Pa, the deposition rate of the C:H:N:O film inside the aggregation chamber approached zero (Figure 2a). At this moment, the C:H:N:O NPs became detectable by the quartz crystal microbalance (QCM) that was inserted into the main deposition chamber. This suggests that starting at a pressure of 100 Pa, the inter-molecular collisions prevented the out-diffusion of the sputtered fragments away from the plasma and forced them to recombine in the volume of the discharge that gave rise to the formation of the plasma polymer NPs [93]. Such formed NPs were afterwards transported by the flow of the carrier gas to the main deposition chamber and detected by the QCM. A further increase of the pressure subsequently led to an increased deposition rate of the C:H:N:O NPs. However, as can be seen in Figure 2b, the deposition rate was found to not be temporally stable—instead of a linear rise of the frequency shift of the QCM (the shift in the resonant frequency is directly proportional to the mass deposited) with the deposition time, the NPs arrived to the crystal in periodically repeated pulses. The frequency of these deposition bursts was approximately 1 per minute. Such behavior is well-known in the field of dusty-plasma and is connected to the charging of growing NPs, the confinement of negatively charged NPs in a plasma potential, and their subsequent release from the plasma bulk as soon as they reach a critical size [111]. The mono-dispersity of the produced NPs (see Figure 3) suggests that all the NPs reached the critical size at the same time. Furthermore, the critical size of the NPs was highly sensitive to the operational parameters (plasma density, energy of charged species, gas flow, gas temperature, working gas, etc.) and therefore the size distribution of the NPs can be finely tuned in a relatively wide range by adjusting these parameters [89,93]. An example of this behavior is presented in Figure 3, where the NPs produced using either argon or nitrogen are compared. 

**Figure 4:**
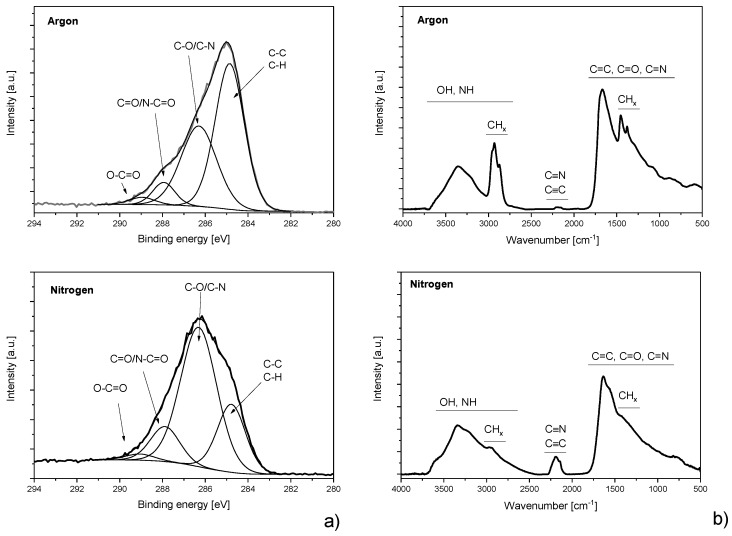
(**a**) High-resolution X-Ray Photoelectron Spectroscopy (XPS) spectra of C 1s peak of NPs deposited in Ar (top) and N_2_ (bottom) measured by XPS (Phoibos 100, Specs) with an Al Kα X-ray source (1486.6 eV, 200W, Specs). (**b**) Fourier Transform Infrared Spectroscopy (FT-IR) spectra of NPs deposited in pure Ar (top) and N_2_ (bottom) recorded by FT-IR (Bruker Equinox 55) in a reflectance-absorbance mode using gold-plated silicon wafers as substrates.

Naturally, the variation of the working gas led not only to the variation of the mean size of the produced NPs but also to the alteration of their chemical composition, in analogy to the deposition of thin films by the RF magnetron sputtering of Nylon [108]. For instance, the substitution of argon by nitrogen resulted in the formation of nitrogen-rich NPs. This is demonstrated in Figure 4, where the high-resolution C 1s X-Ray Photoelectron Spectroscopy (XPS) spectra and Fourier Transform Infrared Spectroscopy (FT-IR) spectra are compared as measured on the NPs deposited with either argon or nitrogen and in Table 1, where elemental composition of C:H:N:O NPs is presented. Evidently, the substitution of argon by nitrogen caused a substantial decrease of the fraction of C-C/C-H chemical bonds that was accompanied by the significant increase of the number of nitrogen-containing moieties. This is a very important and valuable feature as it allows for the production of NPs not only with different sizes but with altered chemical compositions and related functionalities. 

### 3.2. Composite Nylon/Cu Target

**Figure 5:**
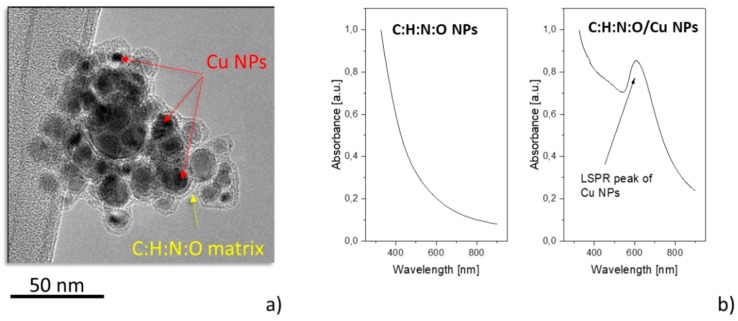
(**a**) Transmission electron microscopy (TEM) image of multi-core@shell Cu/C:H:N:O NPs produced when the composite target was used. (**b**) UV-Vis spectra of C:H:N:O NPs and heterogeneous Cu/ C:H:N:O NPs. RF power 80 W.

The first strategy tested with the aim to produce heterogeneous metal/C:H:N:O NPs was based on the utilization of a Cu/Nylon composite target. It was found out that the introduction of the Cu strip onto the Nylon 6,6 target resulted in the formation of composite NPs at a relatively high pressure, which assured the stable production of bare C:H:N:O NPs. However, the supplied RF power had to be increased up to 80 W to provide a sufficient supply of copper. As shown in Figure 5a, NPs produced in this way had a rather complicated structure—the NPs were composed of Cu particles with different sizes (from several nm up to almost 50 nm) that were all embedded into a plasma polymer matrix. Such a structure with multiple Cu cores enveloped by a shell of the plasma polymer resembles the structure of NPs formed when the metallic target was sputtered in the Ar/hexamethyldisiloxane mixture [112]. It is assumed that the multi-core@shell NPs originated from the competing growth of metallic and plasma polymeric NPs, plasma polymerization, phase segregation of the metal and plasma polymer, and subsequent coalescence of the produced heterogeneous NPs.

The presence of metallic NPs was also evidenced by UltraViolet-Visible (UV-Vis) spectroscopy that showed a localized surface plasmon resonance (LSPR) peak at about 600 nm, which is typical for Cu NPs embedded in plasma-sputtered nylon [66] (Figure 5b). 

**Figure 6:**
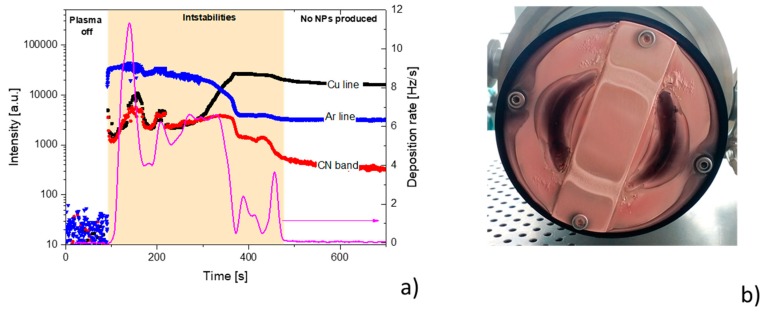
(**a**) Time evolution of intensities of spectral emission lines and bands of Ar, Cu, and CN measured by emission spectrometer (AvaSpec 3648, Avantes) together with the deposition rate of produced NPs. (**b**) Photography of Nylon/Cu composite target after the plasma operation. RF power 80 W.

However, the deposition process was found to be very unstable. After switching on the plasma, the deposition rate initially fluctuated, with a frequency close to the one observed for the case in which only the Nylon 6,6 target was used. This changed after several minutes of the plasma operation when the production of heterogeneous Cu/C:H:N:O NPs dramatically decreased and eventually completely stopped (Figure 6a). The aforementioned temporal evolution of the deposition rate followed the same trend as the intensities of Ar (750 nm) and CN (B^2^Σ^+^−X^2^Σ^+^ at 388 nm) spectral emission lines and bands. The most noteworthy was the substantial decrease of the intensities of these spectral systems after approximately 3 min of the plasma operation, i.e., at the time at which the production of the NPs started to decrease rapidly. In contrast, the intensity of Cu spectral lines was found to significantly increase 3 min after the plasma ignition, which suggests enhanced sputtering of copper at the later stages of the plasma operation. The enhanced sputtering of Cu was accompanied by a partial re-deposition of Cu back onto the target that limited sputtering of its polymeric part. Indeed, the substantial re-deposition of copper was confirmed by a visual inspection of the target after the magnetron operation, which showed that almost all the target’s surface had been covered by copper (see Figure 6b). Because of this, the target had to be dismounted and cleaned in order to restart the production of Cu/C:H:N:O NPs, which limits the applicability of this deposition strategy. Furthermore, the high power necessary for the efficient sputtering of copper resulted in the substantial heating of the system at longer plasma durations (The temperature of the target approached 150 °C after 5 min of the plasma operation as measured by IR-thermocamera). The elevated temperature subsequently hindered the nucleation of NPs and thus no NPs were formed or detected after the prolonged plasma operation. 

### 3.3. System with Two Independent Magnetron

**Figure 7:**
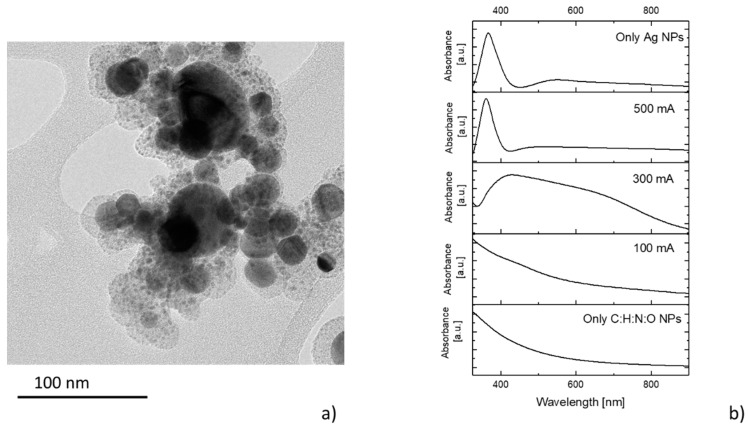
(**a**) TEM image of NPs produced when the dual-magnetron Gas Aggregation cluster Source (GAS) system was used. (**b**) UV-Vis spectra of produced NPs at different DC magnetron currents used for silver sputtering. RF power 40 W.

In order to avoid the issues connected with the gradual covering of the Nylon part of the target by the metal re-deposit, two independent magnetrons were installed in the aggregation chamber (Figure 1c). In this situation, both the high pressure and the direction of the gas flow limited the contamination of the Nylon target by the metallic layer (silver in this case). As a result, the production of the NPs became temporally stable. Nevertheless, the synthetized NPs also exhibited a multi-core@shell structure (Figure 7) similar to the one observed when the composite target was used (Figure 5). Such a finding was expected, as both the plasma polymerization and the growth of the NPs were also running simultaneously in this case. 

In contrast to the setup with the composite Cu/Nylon target, the use of two individual magnetrons offered—besides a better stability of NPs production—higher flexibility in terms of the produced materials, as it allowed for the independent control of the sputtering rates of polymer and metal. In other words, decoupling the sputtering of Nylon and metal made it possible to regulate the metal/plasma polymer ratio in the produced NPs. This effect is demonstrated in Figure 7b, where UV-Vis spectra are presented for the samples that were prepared at a constant RF power of Nylon sputtering, but at different DC magnetron currents for the sputtering of silver. For the low DC, the UV-Vis spectra had a shape similar to the spectra of the C:H:N:O NPs, with only a weak LSPR peak of silver located approximately at 450 nm. With the increasing DC magnetron current, i.e., with the increasing amount of sputtered silver, the intensity of the LSPR peak also increased, which reflects the higher number of Ag NPs embedded into the C:H:N:O matrix. In addition, the width of the silver LSPR peak dramatically broadened due to the wide size distribution of the silver inclusions in the “nanocomposite” NPs. Finally, for the magnetron current of 500 mA, the UV-Vis spectrum resembled the one obtained when only Ag NPs were produced with the RF magnetron switched off. In other words, the production of Ag NPs started to dominate over the production of the plasma polymer matrix and the NPs were mostly metallic. The absence of the plasma polymer matrix/envelope also caused a hypsochromic shift of the silver LSPR peak to 370 nm.

### 3.4. In-Flight Coating of C:H:N:O Nanoparticles

**Figure 8:**
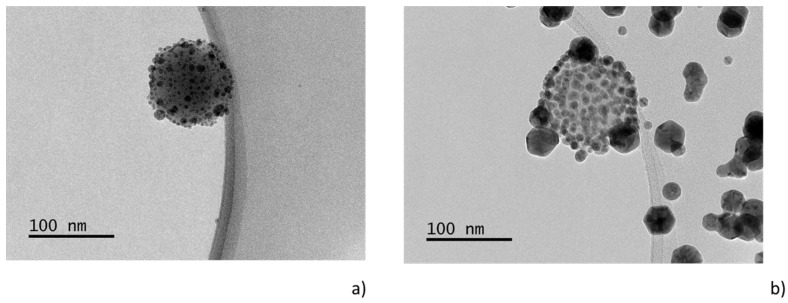
TEM images of Ag/C:H:N:O NPs produced by in-flight sputter deposition of silver onto C:H:N:O NPs. DC magnetron current for silver deposition (**a**) 100 mA and (**b**) 300 mA. RF power 40 W.

In the two previous cases, i.e., for the situations when either the composite target or the dual-magnetron system was used, multi-core@shell NPs were produced due to the fact that both the plasma polymerization and the production of NPs took place at the same time. In order to fully decouple these processes, a system for the in-flight coating of NPs was recently developed [113]. In this case, the gas-phase production of C:H:N:O NPs was separated from the deposition of metal (silver) that was performed in the auxiliary “inoculation” chamber. As a result, the final structure of the NPs differed significantly; instead of multi-core@shell NPs, C:H:N:O NPs decorated by small Ag nanoparticles were formed, as depicted in Figure 8. Such strawberry-like structures arose as a result of the condensation of supersaturated silver vapor on the C:H:N:O NPs that acted as efficient sites for the nucleation of silver NPs. Silver atoms were adsorbed onto the surface of the C:H:N:O NPs and subsequently formed small Ag NPs in a similar way to the “conventional” sputter deposition of metals onto solid substrates at lower pressures. However, the drawback of this configuration is that the volume growth of Ag NPs was not fully inhibited, thus purely silver NPs were formed alongside the metal/plasma polymer strawberry-like NPs. Such metallic NPs, which are considerably bigger than those detected on the surface of the C:H:N:O nanoparticles, are clearly visible in Figure 8, especially for the higher DC magnetron currents used for silver sputtering. 

These preliminary results, which show that the strawberry-like metal/plasma polymer NPs can be prepared in a fully physical way, are very promising, as this method may substitute recently used techniques that employ wet-chemical synthesis. Further research is, however, still needed in order to enable the production of nanoparticles with tailor-made properties (e.g., amounts of metallic NPs attached to a single plasma polymer NPs). 

## 4. Conclusions and Outlook

This featured article summarizes the principles and different strategies that utilize magnetron-based gas aggregation cluster sources for the fabrication of heterogeneous metal/plasma polymer nanoparticles. In comparison with recent results reported for metal-metal NPs, this article shows that three different strategies may be followed: (i) the use of a metal/polymer composite sputtering target, (ii) a multi-magnetron strategy, and (iii) in-flight deposition of metal onto plasma polymer NPs. It was shown that depending on the followed strategy, NPs with different structures can be produced. For the cases in which the plasma polymerization and formation of both metal and plasma polymer NPs took place at the same time, multi-core@shell nanoparticles were produced. From this point of view, the procedure that utilizes two independent magnetrons for sputtering of metal and polymer targets allows for the tailoring of the properties of the formed nanoparticles, which is not possible when a single composite target is used. Furthermore, the results reveal that the complete decoupling of plasma polymer NPs production and the sputtering of metal opens the way to synthetizing core-satellite NPs, i.e., nanoparticles, with a large plasma polymer core decorated by numerous small metallic NPs. Although emphasis was placed solely on metal/C:H:N:O NPs in this study, it is worth stressing that similar procedures may be employed for other combinations of materials. Furthermore, the physical method of NPs production reported here offers several key benefits as compared to methods based on the chemical synthesis of heterogeneous NPs and thus presents a vivid alternative to them. Nevertheless, it is also important to note that many challenges still have to be faced before the wider spread of such nanomaterials in various fields (e.g., bio-sensing, tissue engineering, drug delivery). These challenges are related to a better control of the physico-chemical and/or bio-related properties of produced NPs, which requires not only more targeted experiments using different materials/configurations/operational conditions but a better understanding of the processes that occur during the formation and growth of NPs. Thus, to conclude, the presented results should be considered as a promising starting point that opens new opportunities for the sputter deposition of functional nanomaterials.

## Figures and Tables

**Table 1:** Elemental composition of C:H:N:O NPs deposited using Ar or nitrogen and relative contributions of different bond types, resulting from spectral de-convolution of C 1s peak.

Working Gas	O [at.%]	C [at. %]	N [at. %]	C-C/C-H [%]	C-O/C-N [%]	C=O/N-C=O [%]	O-C=O [%]
Ar	12	76	12	55	37	6	2
N_2_	6	64	30	23	62	12	2
